# Immunomodulation in Pediatric Asthma

**DOI:** 10.3389/fped.2019.00289

**Published:** 2019-07-12

**Authors:** Amelia Licari, Sara Manti, Riccardo Castagnoli, Alessia Marseglia, Thomas Foiadelli, Ilaria Brambilla, Gian Luigi Marseglia

**Affiliations:** ^1^Department of Pediatrics, Fondazione IRCCS Policlinico San Matteo, University of Pavia, Pavia, Italy; ^2^Unit of Pediatric Genetics and Immunology, Department of Pediatrics, University of Messina, Messina, Italy; ^3^Department of Clinical and Experimental Medicine, University of Catania, Catania, Italy

**Keywords:** asthma, children, endotypes, biologics, treatment, omalizumab, mepolizumab

## Abstract

Childhood asthma is actually defined as a heterogeneous disease, including different clinical variants and partially sharing similar immune mechanisms. Asthma management is mainly focused on maintaining the control of the disease and reducing the risk of adverse outcomes. Most children achieve good control with standard therapies, such as low doses of inhaled corticosteroids (ICS) and/or one or more controller. These medications are targeted to suppress bronchial inflammation and to restore airway responsiveness. However, they are not disease-modifying and do not specifically target inflammatory pathways of asthma; in addition, they are not significantly effective in patients with severe uncontrolled asthma. The aim of this review is to update knowledge on current and novel therapeutic options targeted to immunomodulate inflammatory pathways underlying pediatric asthma, with particular reference on biologic therapies.

## Introduction

Asthma represents a major health problem in the pediatric population worldwide. Childhood asthma is actually defined as a heterogeneous disease, including different clinical variants (phenotypes) and partially sharing similar immune mechanisms (1). Asthma management is mainly focused on maintaining the control of the disease and reducing the risk of asthma-related exacerbations and deaths (2). Most children achieve good control with standard therapies, such as low doses of inhaled corticosteroids (ICS) and/or one or more controller (2). These medications are targeted to suppress bronchial inflammation and to restore airway responsiveness. However, they are not disease-modifying and the inflammation return on their discontinuation; in addition, they are not significantly effective in patients with severe uncontrolled asthma (3, 4).

A number of individualized therapies, such as biologics, are available as add-on treatment in adult asthmatic patients with severe uncontrolled symptoms (5). When considering children, only few biologics have been approved and there is limited experience in this population (6) (Table 1).

**Table 1:** Biologicals approved for treatment of severe asthma.

**Medication**	**Target**	**Indication for asthma**	**Dosing and administration**	**Efficacy**	**Adverse effects**	**Biomarkers for primary selection of patients**
Omalizumab	IgE	Moderate-severe allergic, age ≥ 6 y	75–600 mg SC q2–4 wk based on IgE and wt	↓ Asthma exacerbations, ↓ symptoms, ↑ FEV_1_, ↑ QoL, ↓ ICS dose, ↓ seasonal exacerbations	Anaphylaxis < 0,2% Headache Pharyngitis Injection site reactions	IgE level 30–1,500 IU/ml sIgE against perennial allergens FeNO> 30 ppb
Mepolizumab	IL-5	Severe eosinophilic, age > 12 y (US), ≥ 6 y (EU)	100 mg SC q4 wk	↓ Asthma exacerbations, ↓ symptoms, ↑ FEV_1_,↑ QoL, ↓ ICS dose	Headache Pharyngitis Hypersensitivity reactions	Blood eosinophils ≥ 150–300 cells/μl
Reslizumab	IL-5	Severe eosinophilic ≥ 18 y (US)	3 mg/kg IV q4 wk	↓ Asthma exacerbations, ↓ symptoms, ↑ FEV_1_,↑ QoL, ↓ ICS dose	↑ CPK (20%) Myalgia (1%) Pharyngitis Anaphylaxis < 1%	Blood eosinophils ≥ 400 cells/μl
Benralizumab	IL-5R	Severe eosinophilic, age ≥ 12 y (US)	30 mg SC q4 wk for the first 3 doses followed by 30 mg q8 wk	↓ Asthma exacerbations, ↓ symptoms, ↑ FEV_1_,↑ QoL, ↓ ICS dose	Hypersensitivity reactions Headache Pharyngitis Injection site reactions	Blood eosinophils ≥ 300 cells/μl
Dupilumab	IL-4R	Moderate-severe eosinophilic, age ≥ 12 y (US)	Initial dose 400–600 mg, then 200–300 mg SC, q2 wk	↓ Asthma exacerbations, ↑ FEV_1_	Anaphylaxis Hypersensitivity reactions Pharyngitis	*not yet defined*

*CPK, creatinine phosphokinase; EU, European Union; FEV_1_, forced expiratory volume in 1 s; ICS, inhaled corticosteroids; IV, intravenously; q, every; QoL, quality of life; SC, subcutaneously; sIgE, specific IgE; US, United States; vs, versus; wk, week; wt, weight; y, years old; ↓, reduction; ↑, increase*.

## Cellular and Molecular Mechanisms in Asthma

Asthma phenotypes are closely related to airway inflammatory pathways (endotypes), which are determined by numerous cell types, mediators, and immune pathways (7, 8). Two major distinct inflammatory endotypes have been recognized so far: T (Type) 2 and non-T2 endotype.

Eosinophilic inflammation is predominant in T2 endotype and is driven by allergy in more than a half of patients (9). When exposed to allergens and/or to microbes and pollutants, airway epithelial cells release cytokine mediators such as interleukin (IL)-33, IL-25, and thymic stromal lymphopoietin (TSLP)—the so called “alarmins”—that initiate multiple signaling pathways (10). While interleukin (IL)-33 and IL-25 mainly activate type 2 innate lymphoid cells (ILC2s), TSLP also primes dendritic cells (DCs) to promote T2 immunity by activating CD4+ Th2 cells and B cells. CD4+ Th2 cells are the principal driver of eosinophilic airway inflammation by generating abundant quantities of IL-4, IL-5, and IL-13 (Th2 cytokines) (7). IL-4 activates B cells to differentiate into plasma cells that generate immunoglobulin E (IgE) required for mast cell responses to allergens; IL-5 promotes eosinophil differentiation and survival; IL-13, IL-4, and other inflammatory mediators promote goblet cell overexpression, increased mucus secretion, as well as airway hyperresponsiveness, then contributing to the hallmarks of asthma pathophysiology (7, 11–13). In allergic asthma, allergen-specific IgE contributes to the amplification of this inflammatory pathway by inducing a delayed phase reaction characterized by the massive influx of eosinophils and other inflammatory cells (7). Moreover, IgE seems to be directly involved in the pathogenesis of airway remodeling, since the expression of its receptors has been recently demonstrated on airway smooth muscle cells (14, 15).

Non-T2 asthma is marked by a neutrophilic cellular infiltrate or few cells (pauci-granulocytic). The neutrophilic inflammation is mainly the results of a mixed Th1 and Th17 cytokine milieu (IL-8, IL-17A, IL-22) triggered by infections and/or inhaled pollutants, while the pauci-granulocytic inflammatory profile is still largely unknown (13). A role of systemic and metabolic inflammation has also been supposed to contribute to non-T2 endotype, since it is prevalent in obese and older patients (16).

This dual endotype categorization has proven to be clinically relevant and, in particular, eosinophilia directly correlates with corticosteroid response, onset of disease and symptoms (17). The identification of the inflammatory endotype and the prediction of a specific treatment response rely on validated non-invasive biomarkers, such as eosinophils (in blood and sputum), IgE, fractional exhaled nitric oxide (FeNO), and serum periostin, which are available in clinical practice and are related to T2 asthma endotype (7, 17–19). Even though both pathways may coexist in some few patients, the T2 endotype is found in the majority of asthma patients, in particular in children (7). Thus, the development of novel biologic treatments has been focused on this component of the inflammatory pathways.

## Immunomodulating Approaches in Pediatric Asthma

One of the primary aims of asthma treatment is the reversal of existing airway inflammation, hence, the therapeutic strategies have focused on either reducing inflammatory cells and mediators or blocking their effects.

The local immune effects of ICS in asthmatic airways include anti-inflammatory gene activation and switching off inflammatory gene expression which affect the synthesis of inflammatory and anti-inflammatory cytokines/chemokines, receptors, enzymes, and adhesion molecules, and result in decreased inflammatory cell survival and recruitment (20). Moreover, ICS increase the β2-receptor expression, function, and signaling, thus, prevent the development of tolerance to β2-agonists in asthmatic patients treated with β-agonist bronchodilators (21). ICS also act by decreasing vascular permeability and the release of secretagogue from macrophages, reducing local edema and mucus secretion. Finally, the prevention of the cytotoxic effect of the major basic protein (MBP) released from eosinophils is the most common ICS-mediated eosinopenic effects (20).

However, the ICS fail to inhibit leukotriene-induced airway inflammation, thus, the use of leukotrienes (LT) modifiers can be crucial in asthma management, offering additional clinical benefit. Two different types of LT modifiers have been identified: LT synthesis inhibitors and cysteinyl leukotriene receptor (CysLT) antagonists. By interrupting the 5-lipoxygenase pathway, the LT synthesis inhibitors hinder the synthesis of all leukotrienes. The CysLT antagonists influence the bronchoconstrictor and pro-inflammatory activity of cysteinyl leukotrienes (LTC4, LTD4 LTE4) within the asthmatic airway (22).

Novel interventional approaches to modulate the pathogenic immune response have demonstrated significant benefits in preventing the development of asthma and in treating established asthmatic disease (23).

### Allergen Immunotherapy

Currently, allergen immunotherapy (AIT) is the only disease-modifying treatment strategy for allergic patients (24). It is proven to be the only therapy that alters the natural history of allergic disease, prevents its progression and the development of new sensitizations and may even delay the development of asthma in patients with allergic rhinitis (25). AIT demonstrated to induce a persistent immunological and clinical tolerance toward the causal allergen, through molecular mechanisms involving both innate and adaptive immunity (26–28). In particular, AIT upregulates allergen-specific T-regulatory (Treg) cells and B-regulatory (Breg) cells, inhibiting the activation of CD4+ Th2 lymphocytes, suppressing allergic inflammation and shifting toward a Type 1-mediated immune response, releasing cytokines, interleukin (IL)-10 and transforming growth factor-β (TGF-β) (27, 29–31). Several studies showed a significant reduction in asthma symptoms, reduction in use of medications and improvement in bronchial hyperreactivity following AIT (32). In children, randomized clinical trials (RCTs) and meta-analyses confirmed the clinical effectiveness of AIT in asthma, possibly even in long-term (33, 34); AIT may also contribute to delay or prevent the onset of asthma in children (35–37). More recently, after the positive clinical results of a Phase III clinical trial evaluating the treatment of asthma with standardized quality (SQ) house dust mite (HDM) sublingual (SLIT)-tablet, GINA (Global Initiative for Asthma) endorsed this specific SLIT product in adolescents and adults with mild-to-moderate and controlled HDM-asthma (2). According to this recommendation, severe asthma represents a clinical contraindication for AIT (2, 38). Although biologics in severe asthma and AIT in allergic diseases target two different populations, biologic therapies have been coupled to AIT to treat asthmatic patients at high risk of adverse reactions in a novel experimental therapeutic approach (39).

### Biologic Therapies

The development of the biological drugs has revolutionized the therapeutic approach to asthma, particularly in patients with severe disease and resistant to standard treatment. These drugs are characterized by an innovative and highly selective mechanism of action, based on the targeted inhibition of specific molecular or cellular targets directly involved in the pathogenesis of airway inflammation (5).

## Biologics for T2 Asthma in Children

### Anti-IgE

The pharmacological blockade of IgE represents a milestone in the field of biologic treatments for severe asthma (40). Omalizumab is the first available humanized monoclonal anti-IgE with the pediatric indication (age ≥ 6 years) for severe asthma (41). It is indicated as add-on treatment for children with severe allergic asthma with elevated serum IgE (>30 and <1,500 IU/ml) and serum IgE positivity for at least one aeroallergen (42). Omalizumab is recommended to be administered as a subcutaneous (SC) injection every 2–4 weeks based on body weight and serum IgE level (43). After binding circulating IgE, omalizumab decreases IgE levels, inhibits IgE binding with its receptors, and downregulates the expression of IgE receptors on mast cells, basophils and dendritic cells (41). Overall, this results in decreased release of inflammatory mediators related to the allergic response. Several RCTs consolidated the efficacy and safety of omalizumab in the pediatric population (44–47), leading to its final registration more than 10 years ago. Omalizumab demonstrated to be effective in reducing the number of asthma exacerbations requiring oral corticosteroids (OCS), and the need of hospitalizations in severe asthmatic children; these effects resulted in improvement of asthma control and quality of life of these children and their families (48). A significant decrease in the number of seasonal exacerbations triggered by respiratory viruses has been also recently reported in treated subjects, probably due to the restoration of antiviral defenses (in particular type I interferon production) (47, 49, 50). Observational studies conducted in children with poorly controlled asthma demonstrated a significant improvement in asthma control as well as a huge decrease in exacerbation and hospitalization rates over 2 years of therapy (51–54); the impact of this biologic was also observed on the discontinuation of daily OCS, the decrease of ICS dose and a slight improvement of lung function (51–54). Safety data derived from clinical trials, observational studies and post-marketing analyses showed that omalizumab is characterized by a very good profile of safety and tolerability in children and adolescents (55–59). In particular, injection site reactions, usually of mild-to-moderate severity and short in duration, were the most reported side effects (55, 56); anaphylactic events have not been observed in pediatric studies, unlike those in adults and adolescents (51–53, 59). Finally, there is no evidence to support an increased risk of malignancy in patients treated with omalizumab (57, 58); however, a long-term monitoring of treated patients is still required to confirm the good safety profile.

Despite the widespread clinical use of omalizumab in the pediatric population, a number of questions remain unanswered based on available scientific data. The profile of the best responding patient phenotype has not been identified yet: having severe asthma with multiple allergic comorbidities associated with raised blood eosinophil count, high levels of total IgE and fractional exhaled nitric oxide (FeNO) seem to be predictive of a positive clinical response in the pediatric population (60, 61). Age < 6 years, IgE > 1,500 IU/ml, and non-allergic severe asthma together represent the current limit for omalizumab use in children, as well as in adolescents and adults. Preliminary studies have been conducted in non-allergic children (62) and children with excessively high IgE levels (63) with positive encouraging results; a single study on uncontrolled asthmatic children <6 years is actually ongoing (Preventing Asthma in High Risk Kids study, NCT02570984) with the aim to evaluate the disease-modifying effect of anti-IgE therapy. The optimal duration of omalizumab therapy has not been determined, but it is considered an effective treatment approach to continue treatment in responders for at least 2 years, based on observational data in children (64, 65). Finally, its long-lasting effect after suspension has been not yet clearly defined. The definition of targeted courses of therapy may represent the starting point for optimizing the cost-effectiveness of this biologic treatment in the pediatric population.

### IL-5

Mepolizumab, a murine humanized IgG1 monoclonal antibody, was the first anti eosinophil-targeted molecular therapy to be validated in patients with severe asthma. Mepolizumab acts against circulating IL-5, preventing the IL-5/IL-5Rα interaction on the surface of eosinophils, and, thereby, affecting the release and growth of eosinophils (66).

In 2015, mepolizumab has been approved as add-on maintenance therapeutic option for the treatment of severe eosinophilic asthma in patients who are 12 years and older, and then in pediatric population 6 years old and above (67). The recommended dose of mepolizumab is 100 mg for adults and children older than 12 years of age, and 40 mg for children aged 6 to 11 years old, both administered subcutaneously (66, 67).

Mepolizumab has demonstrated favorable efficacy profile in decreasing the number of asthma exacerbations, improving lung function, asthma control and quality-of-life (QoL) scores, as well as significantly reducing OCS use (68–70). Interestingly, all these outcomes were maintained over time, although, lung function, expressed in terms of forced expiratory volume in 1 s (FEV1), was gradually decreasing to approximately baseline, reflecting a stabilization of lung function over the course of the treatment period (71–73).

Treatment response criteria and duration of therapy have been the subject of considerable debate. Exacerbation rate, OCS treatment, blood eosinophil count, and lung function have been proposed as treatment response criteria (74). Besides, the decision to continue mepolizumab treatment should be annually evaluated and based on assessment of at least 50% reduction in exacerbation frequency (75).

Regarding safety, mepolizumab appeared well-tolerated, with the most commonly described adverse events being injection-site reactions, airway infections, exacerbations of asthma, headaches, and fatigue (69, 70, 72, 76).

There is limited data on the safety of mepolizumab in children. One case of histiocytic necrotizing lymphadenitis and varicella have been reported by Food and Drug Administration (FDA) in postmarket surveillance of adverse events. However, the association between mepolizumab and these two events still remains uncertain (77).

### IL-4/13

IL-4 and IL-13 are crucial Th2 cytokines directly involved in the inflammatory remodeling occurring in the airways of asthmatic patients (78). Ig switching from class M to E antibodies, airway recruitment of eosinophils, basophils, lymphocytes, and monocytes are the principle effects mediated by IL-4 and IL-13. Also, while IL-4 mediates polarization and maintenance of T2-type immune response, IL-13 induces airway goblet cell hyperplasia.

Hence, the possibility of blocking or modulating IL-4 and/or IL-13 aroused great interest among researchers aiming to gain therapeutic benefit in asthma.

Currently, dupilumab is the only available biologic drug targeting both IL-4 and IL-13, approved to treat patients with moderate-to-severe asthma and airway or peripheral eosinophilia (78).

Dupilumab appeared to improve both FEV1 and asthma control as well as to decrease T2-inflammation and asthma exacerbation rate (79, 80). In QUEST trial, patients aged over 12 years with uncontrolled, moderate-to-severe asthma were randomized to receive dupilumab or placebo. Over the 52 week treatment period, dupilumab significantly reduced the severe asthma exacerbations rate, especially in patients showing higher baseline eosinophilia (>300 cells/mm^3^) and FeNO values major than 25 ppb (81). The Liberty Asthma VENTURE (82) demonstrated the effectiveness of dupilumab in reducing OCS use in 210 patients with CS-dependent severe asthma (dupilumab groups:placebo group = −70.1%:−41.9%, respectively). Moreover, 48% of patients in dupilumab group completely discontinuing OCS use.

In April 2017, a randomized, double-blind, placebo-controlled, parallel group study to evaluate the efficacy and safety of dupilumab in children 6 to <12 years of age with uncontrolled persistent asthma has been started and not yet concluded (83).

Recently, a systematic review by Zayed et al. evaluated the results of four placebo controlled RCTs, assessing dupilumab safety (84); injection site reactions were commonly described in experimental group. A transient blood eosinophilia was also recorded but it was not associated to any consequence or adverse effect.

Biologics directed exclusively against IL-4 or IL-13 have been also investigated, however both pitrakinra (anti-IL-4R) (85) and tralokinumab (86) and lebrikizumab (anti-IL13) (87) failed to show consistent benefits for the treatment of severe and uncontrolled asthma.

## Biologics for non-T2 Asthma in Children

### Anti IL-25

The evidence of IL-25-mediated Th2 cell differentiation, increase in production of IL-4, IL-5, and IL-13, elevated IgE and IgG levels, eosinophil infiltration, goblet cell hyperplasia and mucus hypersecretion, provided the proof for the role of IL-25 into asthma pathogenesis (88–90). IL-25 is a Th2 cell-derived cytokine belonging to IL-17 family. Following its release from mast cells, eosinophils, basophils, and alveolar macrophages, IL-25 binds IL-17 receptor and induces Th2 cell-mediated inflammatory response in the airways. In a *vivo* model, IL-25 was able to cause airway inflammation and remodeling as well as hypersensitivity (89, 91). By blocking IL-25, a significant prevention of airway hyperresponsiveness and a minor eosinophil infiltration into the lung tissue as well as goblet cell hyperplasia were also noted (89).

However, neutralizing IL-25 activity showed partial efficacy into modulate the airways smooth muscle (91).

To date, no clinical trials are investigating the potential role of anti-IL-25 in asthmatic patients.

### Anti IL-33

Bronchial epithelial cells are also considered the primary source of IL-33, an IL-1-like epithelial-derived cytokine. In response to infectious or inflammatory stimulus, IL-33 binds its receptor ST2 on mast cells; and it stimulates both the Th2-associated cytokines release as well as the Th2/IL-31 and Th17 axis (92). Moreover, acting synergistically with other cytokines such as TSLP and IL-17, IL-33 can induce a pulmonary inflammation, which was found to be glucocorticoid-resistant (93). The critical role of IL-33 was confirmed by GWAS studies showing that IL-33 and ST2 genes were significantly associated with asthma (94). Also, sputum IL-33 values reflected disease severity; higher IL-33 levels were detected in patients with more severe disease (95).

Currently, one phase 1 trial [AMG 282 (RG 6149)] and one phase 2 trial (ANB020) are ongoing in patients affected by asthma (96).

### Anti Thymic Stromal Lymphopoietin (TSLP)

Following inflammatory or infectious injury, and/or allergen exposure, lung derived epithelial cells, airway smooth muscle cells, mast cells, macrophages, granulocytes, and dendritic cells, release TSLP, a cytokine belonging to IL-2 family. Via interaction with its receptor, TSLP amplifies the Th2 polarization causing airway and blood eosinophilia, cells recruitment (mast cells, basophils, and dendritic cells), differentiation of naive T cells into Th2 cells, and proinflammatory cytokines release (97). Several genetic analyses have linked TSLP to Th2-polarized immunity and asthma (98). Bronchial epithelial cells from asthmatics patients express higher TSLP levels than healthy subjects, and, moreover, TSLP expression in the bronchial epithelium and submucosa was correlating with basal membrane thickness, thus, also with disease severity (99).

In a double-blind, placebo-controlled study, 31 patients (age range, 8 to 60 years) with mild asthma were randomized to undergo to 3 monthly doses of AMG 157, a human anti-TSLP monoclonal IgG2, or placebo treatment for 12 weeks. When compared to placebo group, AMG 157 group reported a significant decrease in allergen-induced bronchoconstriction and in systemic and airway inflammation (100). Successively, a phase 2, randomized, double-blind, placebo-controlled trial, enrolling adult patients affected by mild to moderate uncontrolled asthma assessed the efficacy and safety of tezepelumab (AMG 157/MEDI9929), an human IgG2 monoclonal antibody. Tezepelumab administration was associated with a minor annualized asthma exacerbation rate and g a higher increase in prebronchodilator FEV1 (101). The percentage of mild to serious adverse events was similar among experimental and placebo arms (101).

To date, a new clinical trial evaluating the effects of anti-TSLP in adult patients with asthma (UPSTREAM) is ongoing (102).

### Anti IL-17 and Anti-tumor Necrosis Factor (TNF)-α

Several studies demonstrated that IL-17 family of cytokines actively contributes to airway inflammation in non T2 asthma (13). In particular, airway concentration of IL-17 and its related cytokines (IL-17A and IL-25) are upregulated in patients with uncontrolled asthma (13); their levels have been positively correlated to neutrophilic inflammation and asthma severity (13, 103, 104). High levels of serum IL-17 have been also detected in children with asthma and, together with IL-17+ T cells, have been associated with asthma severity in children (105, 106). Likewise, levels of Tumor Necrosis Factor (TNF)-α are increased in either the blood or sputum of patients with neutrophilic asthma, exerting major biological effects on airway inflammation, remodeling, and hyper responsiveness (107, 108). These patients experience persistent symptoms and are prone to frequent exacerbations, which better respond to antibiotics (such as macrolides) rather than to corticosteroids (109). Accordingly, therapeutic strategies to modulate neutrophilic function have been proposed to improve clinical outcomes in non T2 asthma. Cytokine-targeted strategies inhibiting IL-17 and TNF-α receptor signaling both failed to be effective in asthma treatment. Brodalumab (AMG 827), a human anti-IL-17 receptor A monoclonal antibody, demonstrated marginal therapeutic benefit in two Phase 2 studies conducted in adult patients with moderate to severe asthma (110, 111). Golimumab (CNTO148), a human monoclonal antibody against TNF-α, failed to achieve significant treatment effect and demonstrated an unfavorable risk-benefit ratio in adult patients with severe asthma (112).

No ongoing trials are available in adolescents and in children with neutrophilic severe asthma.

## Conclusion

Novel biologic therapies are available as add-on treatment for severe and uncontrolled asthma in adult population. However, when considering special patient populations, such as children, limited treatment options have been approved, as omalizumab and mepolizumab. Moreover, uncertainties regarding optimal treatment duration, ability to modify the disease course, approach to discontinuation, and long-lasting effects still remain unsolved. These gaps are deeper in non-T2 asthma for which the clinical development of the biologic drugs is still in primeval stage. Finally, the wide interpersonal variability in response to biologic treatment confirms the complex mechanisms underlying asthma and lets hypothesize that probably not a single biologic but a “cocktails” of biologics could be a more appropriate treatment approach, providing the possibility to block or influence two or more key pathways, thus, representing a novel and promising strategy to immunomodulate asthma.

## Author Contributions

All authors made substantial contribution to the conception of the work. RC, AM, TF, and IB reviewed the literature on the subject. AL and SM drafted the final version of the manuscript. AL, SM, and GM revised it critically for important intellectual content. All authors finally approved the version to be published and agreed to be accountable for all aspects of the work in ensuring that questions related to the accuracy or integrity of any part of the work are appropriately investigated and resolved.

### Conflict of Interest Statement

The authors declare that the research was conducted in the absence of any commercial or financial relationships that could be construed as a potential conflict of interest.
